# Serotype Distribution, Antibiotic Resistance and Clonality of *Streptococcus pneumoniae* Isolated from Immunocompromised Patients in Tunisia

**DOI:** 10.1371/journal.pone.0140390

**Published:** 2015-10-13

**Authors:** Anis Raddaoui, Alexandra S. Simões, Rekaya Baaboura, Sofia Félix, Wafa Achour, Tarek Ben Othman, Mohamed Béjaoui, Raquel Sá-Leão, Assia Ben Hassen

**Affiliations:** 1 Laboratory of the National Bone Marrow Transplantation Centre, Bab Saadoun, Tunis, Tunisie; 2 Laboratory of Molecular Microbiology of Human Pathogens, Instituto de Tecnologia Química e Biológica António Xavier, Universidade Nova de Lisboa, Oeiras, Portugal; 3 University of Carthage, Faculty of Sciences of Bizerte, Tunis, Tunisie; 4 UR12ES02, Microbiologie de la Greffe de Moelle Osseuse, Université Tunis El Manar, 2092, Tunis, Tunisie; 5 Hematology Ward, National Bone Marrow Transplantation Centre, Bab Saadoun, Tunis, Tunisie; 6 Pediatric Immuno-hematology Ward, National Bone Marrow Transplantation Centre, Bab Saadoun, Tunis, Tunisie; University of Mississippi Medical Center, UNITED STATES

## Abstract

**Background:**

Pneumococcal disease, a major cause of morbidity and mortality globally, has higher incidence among young children, the elderly and the immunocompromised of all ages. In Tunisia, pneumococcal conjugate vaccines (PCVs) are not included in the national immunization program. Also, few studies have described the epidemiology of *S*. *pneumoniae* in this country and, in particular, no molecular typing studies have been performed. The aim of this study was to evaluate serotype distribution, antimicrobial resistance and clonality of *Streptococcus pneumoniae* isolated from neutropenic patients in Tunisia.

**Methods:**

Fifty-nine *S*. *pneumoniae* were isolated from infection (n = 31) and colonization (n = 28) sites of patients (children and adults) attending the National Centre of Bone Marrow Transplantation in Tunis between 2005–2011. All isolates were characterized by serotype, antimicrobial resistance pattern and multilocus sequence typing (MLST).

**Results:**

The majority (66.1%) of the isolates belonged to five serotypes all included in PCVs: 6B, 9V, 14, 19F and 23F. The potential coverage of the 10-valent and 13-valent PCV was of 71.2% and 76.3% respectively. Resistance rates were very high and 69.5% of the isolates were multidrug resistant: non-susceptibility rates to penicillin, amoxicillin and cefotaxime were 66.1%, 40.7% and 27.1%, respectively; resistance rates to erythromycin, clindamycin, tetracycline, chloramphenicol and trimethoprim-sulfamethoxazole, were 69.5%, 61.0%, 37.3%, 22.0% and 67.8%, respectively. The most frequent serotypes had STs characteristic of multidrug resistant international clones known to be highly successful and important causes of pneumococcal infection: Spain 23F-ST81, France 9V/14-ST156, Spain 6B-ST90, 19F-ST320, and Portugal 19F-ST177.

**Conclusions:**

The majority of *S*. *pneumoniae* strains recovered from immunocompromised patients in Tunisia are representatives of multidrug resistant pandemic clones that express serotypes targeted by PCVs. To contain the burden of pneumococcal disease and improve treatment choices among Tunisian immunocompromised patients PCVs should be offered to all of them.

## Introduction

*Streptococcus pneumoniae* remains a major cause of morbidity and mortality worldwide. Pneumococcus is a commensal of the upper respiratory tract of humans. However, it is also a human pathogen responsible for several respiratory tract infections (such as pneumonia) and serious invasive pneumococcal diseases (IPD), such as sepsis and meningitis [1].

Pneumococcal disease has higher incidence among young children, the elderly and the immunocompromised of all ages. Within this latter group, the risk for pneumococcal infection will vary according to the underlying medical conditions but is several fold higher than among healthy people. For example, a study from Canada found that the incidence of IPD among hematopoietic stem cell transplant patients was 30-fold higher than in the general population [2].

During the last four decades *S*. *pneumoniae* strains resistant to antimicrobial agents have been frequently described worldwide and resistance to commonly used antimicrobial drugs such as beta-lactams and macrolides has increased in several countries [3]. In a multicentric study encompassing more than 2000 strains from all continents, rates of penicillin non-susceptibility, of erythromycin resistance, and simultaneous non-susceptibility to both drugs were 33.3%, 22.9% and 16.2% respectively [4]. Non-susceptibility to antimicrobial agents appears to be mostly related with specific serotypes and genetic backgrounds. For instance, multidrug resistance has been associated to serotypes 6A, 6B, 9V, 14, 19A, 19F and 23F [5] and a small number of clones define the majority of the antimicrobial resistant pneumococci [6, 7].

Most *S*. *pneumoniae* strains are covered by a polysaccharide capsule that constitutes the main virulence factor of this pathogen. Although over 95 serotypes have been described, only a few account for the majority of IPD worldwide [8]. Based on that, three multivalent pneumococcal conjugative vaccines (PCV) were developed and became available during the last 15 years: PCV7 (targeting serotypes 4, 6B, 9V, 14, 18C, 19F and 23F), PCV10 (targeting PCV7 serotypes plus serotypes 1, 5 and 7F) and PCV13 (targeting serotypes included in PCV10 and serotype 3, 6A and 19A). A 23-valent polysaccharide pneumococcal vaccine (PPV23) has been available since the 1980’s and targets 12 serotypes included PCV13 and 11 additional serotypes [9].

PCVs were initially licensed for young children but their use for other ages has now been approved in several countries and their routine use in patients with immunocompromised conditions has been recommended [9, 10].

In Tunisia, the first pneumococcal conjugate vaccine, PCV7 became commercially available in 2008, PVC10 and PVC13 became available in 2012–2013. However, due to their high price, they are rarely prescribed and they are not included in the national immunization program. In public hospitals, PPV23 is available for adults at risk of pneumococcal infection.

In Tunisia, a few studies on *S*. *pneumoniae* epidemiology, describing serotype distribution and/or antimicrobial resistance, are available [11, 12] but no study has focused on immunocompromised patients. Also there is no information on the genetic background of Tunisian pneumococcal strains.

In this study we describe the serotype distribution, clonality and antimicrobial susceptibility patterns of *S*. *pneumoniae* strains isolated from infection and colonization sites of patients attending the National Centre of Bone Marrow Transplantation in Tunis.

## Materials and Methods

### Ethics statement

This study was performed with approval from the Local Medical Commitee of Charles Nicolle Hospital, Tunis, Tunisia. As the bacterial strains were analyzed anonymously the study was exempted from Human Research Commitee approval according to the regulations of the Local Medical Ethical Commitee of Charles Nicolle Hospital, Tunis, Tunisia. Specifically, the Medical Ethical Commitee of Charles Nicolle Hospital waived the need for written informed consent.

### Setting and bacterial isolates

The National Center of Bone Marrow Transplantation in Tunis has 60 beds and receives patients with immunodeficiency or blood malignancies, both children and adults. The study took place between June 2005 and December 2011. During this period there were 1837 admissions and 36435 hospitalization days. The average hospital stay was 20.2 days. Systematic sampling was carried out for all patients upon admission and weekly during hospitalization. It included obtaining throat samples, sputum, urine, and stool to identify possible colonizing pathogens. Bacterial pathogens and commensal strains were isolated, identified and antibiotyped. This information is important to guide on an effective first-line antibiotic therapy in neutropenic patients (white blood cells <500) when there is fever or a risk of bacteremia.

The pneumococcal collection included all non-repetitive *S*. *pneumoniae* strains isolated during the study period. Isolates were obtained from clinically relevant sources or through the systematic sampling of colonization sites. When multiple isolates were obtained from a single patient, duplicates were eliminated on the basis of identical patterns of antimicrobial susceptibility.

### Pneumococcal isolation and identification

Samples were plated in blood agar and incubated at 37°C with 5% CO_2_. For *S*. *pneumoniae* identification putative isolates were analyzed by Gram staining, optochin susceptibility testing, Api 20 Strep system (Biomerieux®), and amplification of *psaA* gene as described by Morrison *et al* [13]. The strains were stored at -70°C in 1ml aliquots of Brain Heart Broth with 10% glycerol.

### Antimicrobial susceptibility testing

Antimicrobial susceptibility was tested by the disc diffusion method on Mueller-Hinton agar supplemented with 5% defibrinated horse blood following the recommendations of the Antibiogram Committee of the French Society for Microbiology [14]. The antibiotics tested were: oxacillin (5μg), levofloxacin (5μg), tetracycline (30UI), chloramphenicol (30μg), vancomycin (30μg), teicoplanin (30μg), erythromycin (15UI), clindamycin (15μg), and trimethoprim-sulfamethoxazole (1.25/23.7μg).

Isolates with a zone size for the oxacillin 5 μg disk <26 mm were screened for penicillin non-susceptibility as recommended [14]. Minimum inhibitory concentrations (MIC) of penicillin G, amoxicillin and cefotaxime were determined by Etest (AB Biodisk, BioMérieux) for all putative penicillin non-susceptible pneumococci (PNSP). The MIC breakpoint used for penicillin G was 0.06–2μg/ml and for amoxicillin and cefotaxime was 0.5–2 μg/ml [14].

The results were interpreted according to the recommendations of the Antibiogram Committee of the French Society for Microbiology [14]. *S*. *pneumoniae* ATCC 49619 was used as control strain in all assays. Multidrug resistance was defined as resistance to at least three antibiotic classes.

### Serotyping

Capsular typing was performed by a combination of multiplex PCRs targeting serotypes/serogroups: 1, 3, 5, 6, 7A/F, 9N/L, 10A, 11A, 14, 15A, 15B/C, 16F, 17F, 18, 19A, 19F, 21, 22F/A, 23, 23A, 23F, 24, 31, 33F/A, 34 and 35F using primers previously described (available at http://www.cdc.gov/streplab/pcr.html) and/or by the Quellung reaction method using type specific antisera (Statens Serum institute, Copenhagen, Denmark) [15].

### Multilocus sequence typing (MLST)

The internal fragments of the seven housekeeping genes *aroE*, *gdh*, *gki*, *recP*, *spi*, *xpt* and *ddl* were amplified by PCR and sequenced as described in the MLST website (http://www.mlst.net/). Sequencing reactions were conducted at Macrogen, Inc. (Amsterdam, The Netherlands). Sequencing analysis was done with DNAStar (Lasergene). The identification of allele number and sequence type (ST) was done at the pneumococcal MLST public database. Clusters of related sequence types were grouped into clonal complexes (CC) using the goeBURST program (http://www.phyloviz.net).

### Statistical analysis

To study association between serotypes and antimicrobial resistance, differences between PCV13 serotypes and non-PCV13 serotypes were analysed with the chi-square test (with Yates correction if appropriate).

## Results

### Bacterial collection

During the study period (2005–2011), 59 non-repetitive *S*. *pneumoniae* strains were isolated (Table 1). The clinical sources of the *S*. *pneumoniae* isolates were sputum (n = 45), blood (n = 9), nose (n = 2), ear (n = 1), eye (n = 1), and throat (n = 1). Invasive pneumococcal disease (IPD) strains were defined as those isolated from a sterile body site (blood, n = 9, 15.3%). Non-IPD strains were defined as strains recovered from eye, ear and sputum (the latter if CFU>10^7^) (n = 22, 37.3%). Colonization strains were defined as those recovered from the nose, throat and sputum (the latter if CFU<10^7^) (n = 28, 47.4%).

**Table 1:** Patient characteristics and isolation of pneumococci.

	No. of pneumococcal isolates according to clinical significance^1^		
	IPD (n = 9)	Non-IPD (n = 22)	Colonization (n = 28)
Age (years)			
0–2	2	0	3
>2–18	2	10	13
>18–64	5	11	11
>65	0	0	0
Sex			
male	5	17	19
female	4	4	8

IPD–invasive pneumococcal disease

^1^For two isolates, patient gender and age were not available.

Males accounted for 69.5% (41/59) of the cases. Six isolates (10.2%) were from young children (<6 years old), 24 (40.7%) from older children (6–18 years old), and 25 (42.4%) were from adults (>18 years old). Only two patients had been vaccinated with the 23-valent pneumococcal polysaccharide vaccine. For two isolates, patient gender, age, and vaccine use was not available.

### Serotype distribution

**Fig 1:**
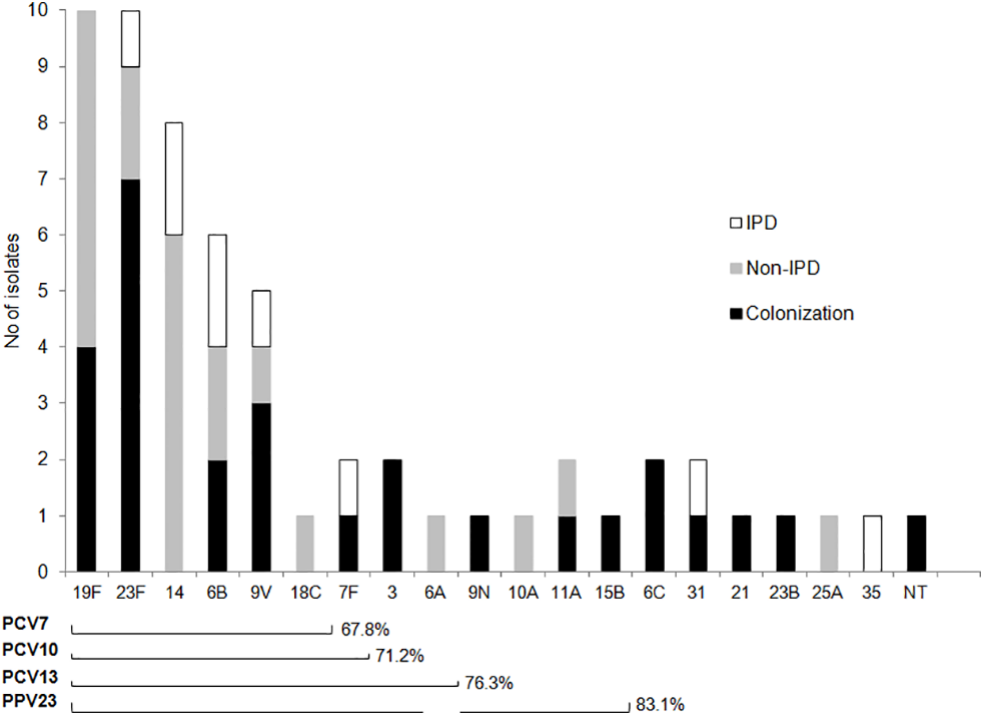
Serotype distribution of *Streptococcus pneumoniae* isolates according to clinical source. IPD, invasive pneumococcal disease; Non-IPD, non-invasive pneumococcal disease; PCV7, 7-valent pneumococcal conjugate vaccine; PCV10, 10-valent pneumococcal conjugate vaccine; PCV13, 13-valent pneumococcal conjugate vaccine; PPV23, 23-valent pneumoccoal polysaccharide vaccine; NT, non-typeable.

Nineteen serotypes and one non-typeable (NT) strain were identified (Fig 1). Two-thirds of the isolates (39 strains or 66.1%) belonged to just five serotypes all included in PCV10 and PCV13: 19F (n = 10), 23F (n = 10), 14 (n = 8), 6B (n = 6), and 9V (n = 5).

The global potential vaccine coverage was 71.2% and 76.3% for PCV10 and PCV13, respectively. Regarding IPD, seven of the nine cases (77.8%) were caused by serotypes covered by PCV10 and PCV13. Most of the 22 cases of non-IPD were caused by serotypes covered by PCV10 and PCV13 (81.2% and 86.4%, respectively). Finally, most colonization strains would be potentially covered by PCV10 and PCV13 (60.7% and 67.9%, respectively). The potential coverage for PPV23 was 83.1%. This included seven of the nine cases of IPD and 20 of the 22 cases of non-IPD.

### Antimicrobial resistance

All isolates were resistant to at least one of the antibiotics tested. Non-susceptibility and resistance rates to penicillin were of 66.1% and 3.4%, respectively (MIC_50_ = 0.075 µg/ml, MIC_90_ = 2 μg/ml). Corresponding values for amoxicillin were 40.7% and 8.5% (MIC_50_ = 0.075 μg/ml, MIC_90_ = 1 μg/ml), and for cefotaxime were 27.1% and 1.7% (MIC_50_ = 0.19 μg/ml, MIC_90_ = 2 μg/ml). Resistance rates to erythromycin, clindamycin, tetracycline, chloramphenicol and trimethoprim-sulfamethoxazole, were 69.5%, 61.0%, 37.3%, 22.0% and 67.8%, respectively. All strains were susceptible to levofloxacin, vancomycin and teicoplanin. Antimicrobial resistance was disseminated among PCV13 and non-PCV13 serotypes being significantly higher for clindamycin, trimethoprim-sulfamethoxazole among PCV13 serotypes (Table 2). Multidrug resistance, defined as resistance to three or more classes of antimicrobial agents, was 69.5% overall, being significantly higher among PCV13 serotypes (Table 2).

**Table 2:** Antimicrobial non-susceptibility of *Streptococcus pneumoniae* isolates.

	No. of non-susceptible isolates (%)			
Antimicrobial agent	PCV13 serotypes (n = 45)	Non-PCV13 serotypes (n = 14)	Total (n = 59)	p-value^1^
Penicillin	35 (77.8)	4 (28.6)	39 (66.1)	0.002
Amoxicillin	21 (46.7)	3 (21.4)	24 (40.7)	0.09
Cefotaxime	14 (31.1)	2 (14.3)	16 (27.1)	0.37
Erythromycin	34 (75.6)	7 (50.0)	41 (69.5)	0.14
Clindamycin	31 (68.9)	5 (35.7)	36 (61.0)	0.03
Tetracycline	17 (37.8)	5 (35.7)	22 (37.3)	0.86
Chloramphenicol	12 (26.7)	1 (7.1)	13 (22.0)	0.21
SXT	35 (77.8)	5 (35.7)	40 (67.8)	0.008
Multidrug resistance	34 (75.6)	7 (50.0)	41 (69.5)	0.14

^1^Chi-square test with Yates correction where appropriate.

### Multilocus sequence typing (MLST)

**Fig 2:**
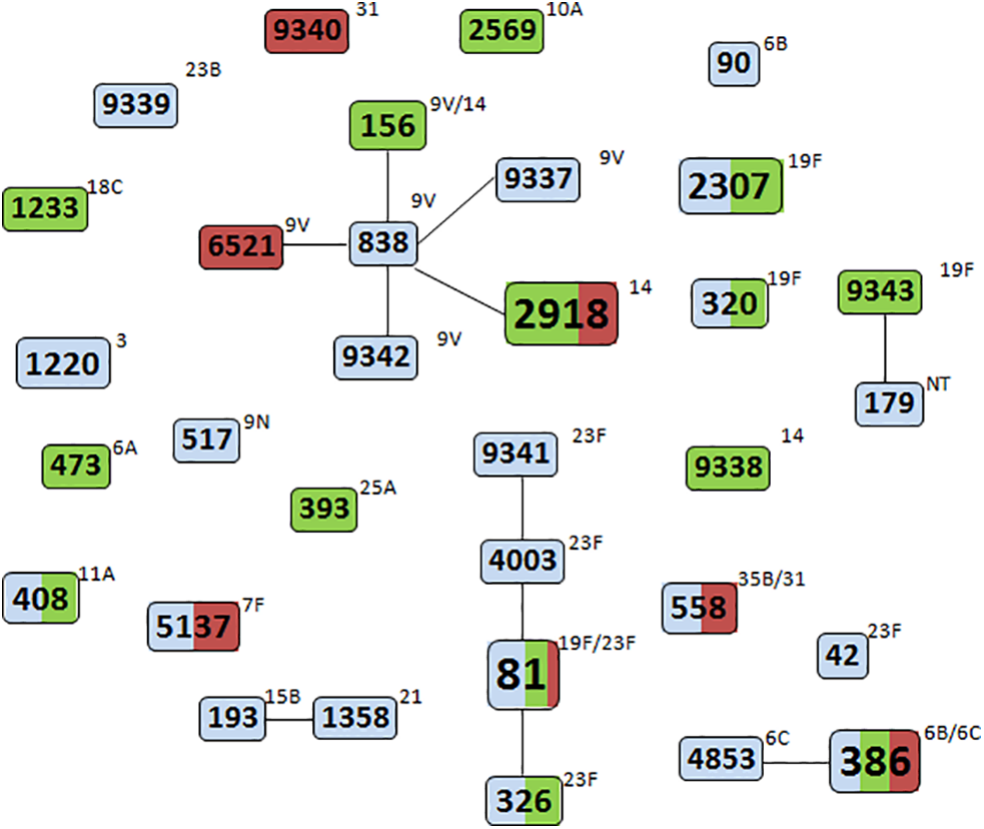
goeBURST representation of *S*. *pneumoniae* isolates. The numbers inside rectangles indicate multilocus sequence types; lines connecting rectangles indicate sequence types that are single locus variants of each other. The size of the rectangle is proportional to the number of isolates displaying the indicated sequence type; the smallest rectangle corresponds to one isolate; the biggest rectangle corresponds to eight isolates. Colors inside rectangles indicate origin of isolates and their relative proportion: blue—colonization isolates; red–invasive disease isolates; green—non-invasive disease isolates. Numbers outside boxes indicate serotype.

The genetic profile of all strains was determined by MLST and 32 sequence types (STs) were identified (Fig 2). Two new alleles (*aroE* 281 and *xpt* 550) and seven novel STs (STs 9337–9343) were found and deposited in the MLST database for *S*. *pneumoniae*. The most frequent STs were ST81 (n = 8), ST386 (n = 6), and ST2918 (n = 6). The two most prevalent CCs were CC838 (with six STs found in 12 strains of serotypes 9V or 14), CC81 (with four STs found in 12 strains of serotypes 19F or 23F) and CC386 (with 2 STs found in 7 strains of serotypes 6B and 6C). No significant relationship between STs/CCs and clinical sources was observed (Fig 2).

MLST results indicated that simultaneous non-susceptibility to penicillin and resistance to erythromycin, although found among isolates of a variety of STs/CCs, was mainly (60%, 21 of 35 isolates) due to isolates belonging to CC81 and CC838. Non-susceptibility to amoxicillin (75%, 18 of 24 isolates) and cefotaxime (69%, 11 of 16 isolates) was also mainly due to CC81 and CC838. Almost all chloramphenicol resistant strains (11/12) belonged to CC81.

## Discussion

In this study we described serotype distribution, antimicrobial susceptibility and clonal properties of *S*. *pneumoniae* strains recovered from disease and colonization sites in immunocompromised patients attending the National Centre of Bone Marrow Transplantation in Tunis, a country where pneumococcal conjugate vaccines (PCVs) are not part of the National Immunization Program. This is the first study from Tunisia looking at the epidemiology of pneumococci among this particular group of patients. It also reports, for the first time, MLST results for pneumococci isolated in Tunisia providing information on the clones in circulation in the country.

We found that the five most prevalent serotypes– 6B, 9V, 14, 19F and 23F - are all covered by current PCVs and, together, accounted for two-thirds of all isolates. These serotypes have also been found to be dominant in a Tunisian pediatric population study [12]. This serotype distribution resembles the one observed in several countries before introduction of PCVs [8] and suggests that, in Tunisia, the pneumococcal population has not been affected by use of PCVs, in agreement with the very low consumption of PCVs in the country. Of interest, we noted the absence of serotype 19A, a serotype that became important in several countries during the last decade, most of which had introduced PCV7 [16, 17]. A low prevalence of this serotype was also noted in the pediatric Tunisian study [12].

In our study, the potential coverage of PCV10 and PCV13 were both high (71.2% and 76.3% respectively). Similar vaccine coverage rates were found in the Tunisian pediatric population (73% and 78%, respectively) [12] highlighting the importance of the introduction of PCVs in Tunisia, not only among young children but also among other risk groups such as the immunocompromised of all ages.

We found high rates of non-susceptibility to antibiotics and of multidrug resistance both among PCV13 serotypes and non-PCV13 serotypes. For example, 66.1% of the isolates were non-susceptible to penicillin. Similar values have been described in other Tunisian studies [12, 18] and contrast with data from other countries such as the neighboring Algeria where a study documented 25.2% of non-susceptibility to penicillin [19]. In Europe, the prevalence of penicillin-non susceptible *S*. *pneumoniae* is considerably lower than in Tunisia ranging between 1–5% in the Netherlands, Norway, Germany, the United Kingdom, Sweden, and Denmark; and c.a. 20% in Ireland, Portugal, Spain, and France [20].

Resistance to macrolides was also very high in our population (69.5%). High values of erythromycin resistance have been described in Asian countries: 96.4% in China, 80.7% in Vietnam and 79% in Sri Lanka [21]. In Europe, resistance levels to macrolides vary significantly but are all much lower: 4–10% in Sweden, the United Kingdom, Denmark, the Netherlands, Norway and Germany; and 20–30% in Portugal, Spain, Belgium, France, and Finland [20].

Resistance rates to other antimicrobials such as amoxicillin (8.5%) and cefotaxime (1.7%) were also high in our study when compared with other countries. For example, in France corresponding rates were of 2% and 0% respectively [22].

These high levels of antimicrobial resistance are alarming and should prompt, per se, additional studies. Antimicrobial resistance has been linked with antimicrobial consumption[23, 24]. In Tunisia, antibiotics can be bought without prescription and this probably leads to overuse and misuse of antibiotics. Currently there are no Tunisian studies correlating resistance rates with antimicrobial consumption. In any case, our results suggest that prompt measures should be taken to contain antibiotic resistance among pneumococci.

To our best knowledge the genetic types of pneumococci circulating in Tunisia have not been described before. MLST data analysis indicated that, despite a high number of STS, including a few novel allelic profiles, most isolates displayed ST previously described. In particular, the most frequent serotypes had STs characteristic of multidrug resistant international clones known to be highly successful and important causes of pneumococcal infection: PMEN clones Spain 23F-ST81 (CC81), France 9V/14-ST156 (CC838), Spain 6B-ST90, 19F-ST320, and Portugal 19F-ST177 [7], http://web1.sph.emory.edu/PMEN/]. The presence of these clones, in particular Spain 23F-ST81 and France 9V/14-ST156, which are associated with serotypes covered by PCV7, explained the majority of both penicillin and resistant strains, chloramphenicol resistant strains and multidrug resistant strains. The expansion of antimicrobial resistance in *S*. *pneumoniae* has been linked with the success of a few multidrug resistant clones [6, 7]. While clones Spain 23F-ST81 and France 9V/14-ST156, were extremely frequent in the 1990’s in several countries worldwide, due to its high fitness, epidemicity, capacity to colonize and cause disease, and multidrug resistance profile [25], introduction of PCV7 has led to its near elimination in many countries [26, 27]. In Tunisia, where PCVs are rarely used, they are still a major cause of concern that could potentially be significantly diminished.

This study has some limitations. First, the number of isolates analyzed was relatively low and originated from a very specific population. This was due to the fact that all isolates were from a single and small health care unit—the Center of Bone Marrow Transplantation in Tunis–which only receives a specific type of patients (immunodeficient or with blood malignancies). This could potentially restrict the scope of our findings not making them general for the rest of the country. Although this may be the case, the fact that several isolates belonged to worldwide epidemic multiresistant clones makes it unlikely that they would be present only in this center and not elsewhere in the country. Another limitation of the study is the absence of data on the prior use of antibiotics, which could potentially bias the pneumococcal collection regarding frequency of antimicrobial resistant isolates. This study has also some strengths as all the available isolates were characterized by three typing techniques: serotyping, antibiotyping and multilocus sequence typing providing useful information that may be used to guide public health measures to contain antimicrobial resistance and pneumococcal disease.

In conclusion, the majority of *S*. *pneumoniae* strains recovered from immunocompromised patients in Tunisia are representatives of multidrug resistant pandemic clones that express serotypes targeted by PCVs. To contain the burden of pneumococcal disease and improve treatment choices among Tunisian immunocompromised patients PCVs should be offered to all of them.
